# abYpap: improvements to the prediction of antibody V_H_/V_L_ packing using gradient boosted regression

**DOI:** 10.1093/protein/gzad021

**Published:** 2023-11-28

**Authors:** Veronica A Boron, Andrew C R Martin

**Affiliations:** Structural and Molecular Biology, Division of Biosciences, University College London, Gower Street, London WC1E 6BT, UK; Structural and Molecular Biology, Division of Biosciences, University College London, Gower Street, London WC1E 6BT, UK

**Keywords:** antibodies, V_H_/V_L_ packing, modeling, prediction, machine learning

## Abstract

The Fv region of the antibody (comprising V_H_ and V_L_ domains) is the area responsible for target binding and thus the antibody’s specificity. The orientation, or packing, of these two domains relative to each other influences the topography of the Fv region, and therefore can influence the antibody’s binding affinity. We present abYpap, an improved method for predicting the packing angle between the V_H_ and V_L_ domains. With the large data set now available, we were able to expand greatly the number of features that could be used compared with our previous work. The machine-learning model was tuned for improved performance using 37 selected residues (previously 13) and also by including the lengths of the most variable ‘complementarity determining regions’ (CDR-L1, CDR-L2 and CDR-H3). Our method shows large improvements from the previous version, and also against other modeling approaches, when predicting the packing angle.

## Introduction

Antibody variability is encoded in the variable fragment (or Fv), which is composed of two protein domains V_H_ and V_L_ from the heavy and light chains, respectively. The V_H_ and V_L_ domains are responsible for antigen binding and are each composed of a framework (two $\beta $-sheets relatively conserved in sequence) and three hypervariable loops (Amzel & Poljak, 1979, Chothia & Lesk, 1987, Chothia *et al.*, 1985, Vargas-Madrazo & Paz-García, 2003). Altering the orientation of the variable domains relative to each other modifies the relative orientation of the CDR loops and thus alters the shape of the binding site (Davies & Metzger, 1983, Naranayan *et al.*, 2009, Novotný *et al.*, 1983); these changes can also affect the elbow-angle (Fernández-Quintero *et al.*, 2020). The V_H_/V_L_ interface interaction can therefore have a direct impact on the affinity with which the antigen is bound (Ben Khalifa *et al.*, 2000, Masuda *et al.*, 2006). Furthermore, the V_H_/V_L_ interaction geometry can determine which of the specificity-determining residues are exposed (and to what degree), and therefore with what type of antigen the antibody is capable of interacting (e.g. haptens, proteins or peptides) (Almagro, 2004, Raghunathan *et al.*, 2012). Thus accurately predicting the interaction geometry of the V_H_/V_L_ interface allows us to get a better picture of the topography of the binding site and potentially modify it during therapeutic design (e.g. during grafting for humanization or for a specific target) (Foote & Winter, 1992).

To date there have been several attempts to tackle the challenge of modeling this interface. Abhinandan and Martin (2010) used residues L35–L38, L85–L88, H36–H39, H89–H92 to define two vectors (one in each domain) from which the V_H_/V_L_ packing angle was determined. A torsion angle between the two vectors was then calculated, averaging -46${}^{\circ }$ with a range of -31${}^{\circ }$ to -61${}^{\circ }$. A genetic algorithm was used to identify residues L38, L40, L41, L44, L46, L87, H33, H42, H45, H60, H62, H91 and H105 as the best predictors of this packing angle. A simple encoding (using four physical properties) of the amino acids at these positions was used to build the machine learning model using a small neural network, with the final result predicting this packing angle with a relative root mean squared error of 0.056. This predictor is referred to as `Predict Antibody Packing Angle' (PAPA). Chailyan *et al.* (2011) also attempted to identify the residues having the most effect on the packing angle. They used a global distance test to calculate the similarity between two structures when a set of the V_H_/V_L_ interface residues (L34, L36, L38, L43, L44, L46, L87, L89, L98, L100, H35, H37, H39, H44, H45, H47, H91, H93, H103 and H105) is superimposed within a certain distance threshold. They looked at 101 V_H_/V_L_ structures and found that their samples clustered into two groups. They then extracted data relating to residues most strongly conserved within their groups and identified a set of residues which they believed to be the most important in discriminating between the groups (L8, L28, L36, L41, L42, L43, L44 and L66).

Further work has been performed since those initial studies. Dunbar *et al.* (2013) expanded the analysis of V_H_/V_L_ packing using the principal component analysis implemented in their ABangle software. The first principal component is similar to the angle defined by Abhinandan and Martin, while the variations in the remaining principal components are fairly small. Consequently, while the approach of Dunbar *et al.* provides a more complete description of the potential variability in packing, the simple single angle defined by Abhinandan and Martin gives a good representation of all major variability. Prediction of packing angle is now part of a number of antibody modeling programs. Bujotzek *et al.* (2015) developed a predictor based on the ABangle descriptor of the V_H_/V_L_ packing and this is now a part of ABodyBuilder2 (Leem *et al.*, 2018). More recently, Weitzner *et al.* published a paper on RosettaAntibody3 (Weitzner *et al.*, 2017), which also incorporates the V_H_/V_L_ packing into its predictions. Currently our own abYmod software (Manuscript in preparation) inherits the packing angle from one of the parent structures, but the packing angle predictor described in this paper is now being integrated into the software.

In this paper, we present an improvement on the Abhinandan and Martin method, using an expanded list of residues and loop lengths as features, as well as improved machine learning methods.

## Methods

### Angle calculation

The packing angle was calculated from the Chothia-numbered Protein Databank (PDB) files using ‘abpackingangle’ available from github.com/ACRMGroup/abpackingangle, which Abhinandan and Martin described in their paper (Abhinandan & Martin, 2010). In summary, the C$\alpha $ positions of eight structurally conserved residues in the light chain (L35–L38, L85–L88) were used to define a vector, with an equivalent vector defined in the heavy chain using residues H36–H39 and H89–H92. A torsion angle is then calculated between the two vectors (about a third vector between the projection of the centroids of the residues onto the light and heavy chain vectors) as a representation of the V_H_/V_L_ region geometry.

### Data preparation

**Fig. 1:**
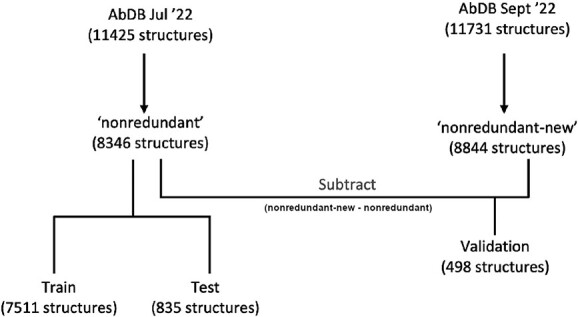
Summary of the file preparation method. The files available from the PDB in July 2022 were used to start. If a file contained multiple antibody structures, the structures were extracted into individual files. Each file then had all the residues of interest summarized with the angle. Redundancy was removed by keeping one file for each set of unique residue–angle pairs. This nonredundant set was then split into test and train sets. Files available from the PDB after July 2022 and until September 2022 were processed in exactly the same way, which created the independent validation set.

Files containing antibodies were obtained from recent local updates to AbDb (Ferdous & Martin, 2018) in July and September 2022. AbDb takes files containing antibodies from the PDB and splits them into individual antibodies (with the antigen, if present). Files are then numbered according to the Kabat, Chothia and Martin numbering schemes and redundant clusters are identified. For the analysis performed here, the Chothia numbered files were used. Those that are not V_H_/V_L_ heterodimers, those having missing residues, those not solved by crystallography and those having resolution worse than 3Å were removed. Starting from this dataset, data preparation is summarized in Fig. 1. The dataset contains both bound and unbound antibodies on the basis that high-affinity antibodies would be expected to show a lock-and-key-type interaction where the packing angle does not change significantly; if binding with antigen has to stress the packing angle away from its unbound optimum, then some of the binding energy will be ‘wasted’ in changing the angle.

To prevent test and training set overlap, but to keep samples where the same key residues produce different angles, the set was non-redundantized based on the residues used as features and the packing angle. Packing angles were rounded to two decimal places and if the residues used as features, and the rounded angle, were identical for multiple structures, only the first sample was kept. This type of non-redundantization was chosen to preserve flexible angles, where the same antibody may have different angles depending on its binding status or crystallization conditions. This was done for tests with both 13 and 37 feature residues. From the data available in July 2022, these structures make up the ‘Nonredundant’ set.

The ‘Nonredundant’ set was then split into ‘Training’ and ‘Test’ sets, the test set being selected as a random 10% of the data and the remaining 90% being used as the training set (Supplementary Files ‘TestSet.txt’ and ‘TrainingSet.txt’).

An independent validation set was prepared by following the same procedure using the files available in September 2022 to create the ‘Nonredundant-New’ dataset (AbDb codes are listed in Supplementary File ‘Nonredundant-New.txt’). The structures available in ‘Nonredundant-New’ (September 2022), but which were not available in ‘Nonredundant’ (July 2022), were extracted to create the ‘independent validation’ set (Supplementary File ‘ValidationSet.txt’).

### Features

**Fig. 2:**
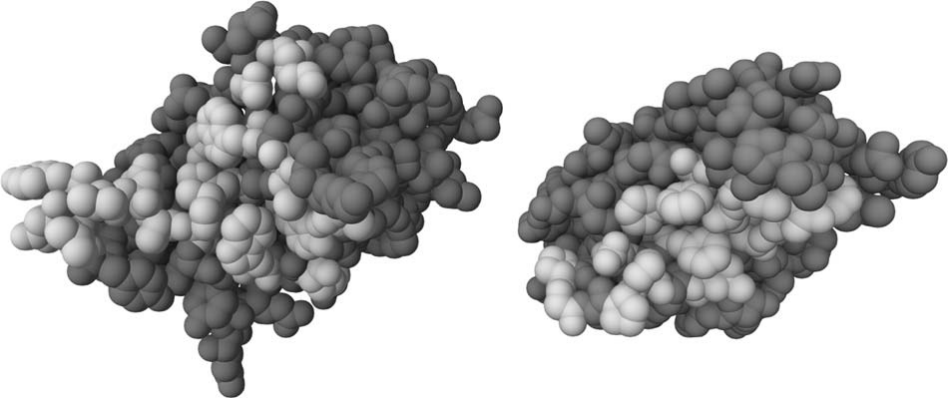
Interface of an antibody Fv (PDB code 5W9K) showing the V_H_ on the left and the V_L_ on the right. One domain should be rotated 180${}^{\circ }$ about the y-axis in order to place it in the orientation that it would adopt when forming an interaction with the other domain. The expanded 37 residue positions used in the predictor (L32, L34, L36, L38, L40, L41, L43, L44, L46, L50, L86, L87, L89, L91, L96, L98, H33, H35, H39, H42, H45, H47, H50, H60, H62, H91, H99, H100, H100A, H100B, H100C, H100D, H100E, H100F, H100G, H103 and H105) are shown in a lighter gray.

Abhinandan and Martin (2010) analyzed the residue positions involved in the V_H_/V_L_ packing and created four sets of positions likely to be the most important on the basis of: (i) highest change in solvent accessible surface area (ASA), (ii) highest average change in ASA, (iii) most frequently occurring positions with highest change in ASA and (iv) most frequently occurring positions with highest average change in ASA. Combining these sets identified a total of 37 residue positions (L32, L34, L36, L38, L40, L41, L43, L44, L46, L50, L86, L87, L89, L91, L96, L98, H33, H35, H39, H42, H45, H47, H50, H60, H62, H91, H99, H100, H100A, H100B, H100C, H100D, H100E, H100F, H100G, H103 and H105, Chothia, Kabat or Martin numbering), highlighted in Fig. 2. They then used a genetic algorithm (where the fitness function was the result of training and testing a neural network) to select the most informative 13 residues (L38, L40, L41, L44, L46, L87, H33, H42, H45, H60, H62, H91 and H105). These two sets (13 residues and 37 residues) were also used in this work.

### Encoding

Abhinandan and Martin encoded the amino acids using a four-physical-parameter encoding scheme (Supplementary File ‘encoding.txt’) which encodes each amino acid using the number of side chain (non-hydrogen) atoms, compactness (number of atoms in the shortest path to the most distal atom), charge (using +0.5 for histidine) and hydrophobicity (using the Eisenberg consensus scale (Eisenberg *et al.*, 1982)). The same encoding was used for all work performed here.

### Re-training the PAPA model (PAPA-Retrained)

The ‘Training’ dataset was used to re-train a neural network identical to that used by Abhinandan and Martin with the feature set of 13 residues as described above. As before, the output values were scaled to a range of 0–1. The neural network consists of a fully connected artificial neural network using an input layer of 13$\times $4 nodes, a single hidden layer of 10 nodes and an output layer consisting of a single real-valued node. Training was performed using Resilient Backpropagation (RProp) (Riedmiller & Braun, 1993) with early-stopping after 150 cycles or a sum-of-squares error $<=1.5$, magnitude pruning and input shuffling. For direct comparison, as before, this was constructed using the Stuttgart Neural Network Simulator (SNNS, www.ra.cs.uni-tuebingen.de/SNNS available at github.com/GunterMueller/SNNS).

### Gradient Boosting Regression model for packing angle prediction

Gradient Boosting Regression (GBR) was implemented using the ‘GradientBoostedRegressor’ estimator of the Python module ‘scikitlearn’. Hyper-parameters are described in Table 1 and were determined by running an extensive grid-search, training on the ‘Training’ dataset and testing on ‘Test’.

**Table 1:** The hyperparameters that were applied when training the gradient boosted regression (GBR) model; these were determined by running a grid search training and testing on the ‘Training’ and ‘Test’ datasets (see the text), respectively.

**parameter**	**value**
alpha	0.01
learning_rate	0.1
max_depth	2
min_samples_leaf	10
n_estimators	50 000
random_state	100

The same four-physical-parameter encoding was used, but here four different feature sets were employed:

- GBR1: The 13 residue feature set used by Abhinandan and Martin,
- GBR2: The 13 residue feature set plus the lengths of CDR-L1 (L24–L34), CDR-H2 (H50–H58) and CDR-H3 (H95–H102),
- GBR3: The full 37 residues identified by Abhinandan and Martin,
- GBR4: The full 37 residues plus the lengths of CDR-L1, CDR-H2 and CDR-H3.

The remaining CDR loops have much less variability in length across antibody structures and it was concluded that their lengths are unlikely to add to the predictive ability.

### Combining Gradient Boosting Classification Regression and Classification

The observed packing angles follow an essentially normal distribution with poorly populated tails. 94% of structures have an angle which is between -40${}^{\circ }$ and -50${}^{\circ }$ and, consequently, predictors can do well by just predicting in this range.

We therefore took an approach of defining this range (-40${}^{\circ }$ to -50${}^{\circ }$) as the ‘normal’ range with anything above that being considered a maximum outlier and anything below considered a minimum outlier.

Thus, an approach which combined classification and regression was tested where these three classes were used to train a gradient boosted classification (GBC) model using the ‘Training’ set. Hyper-parameters were optimized by running an extensive grid-search on the ‘Training’ set and testing on the ‘Test’ set (Table 2). Three separate GBR models were then trained (using hyperparameters in Table 1) on the three separate classes within the ‘Training’ set.

**Table 2:** Gradient Boosting Classification (GBC) hyperparameters, determined by running a grid search training and testing on the ‘Training’ and ‘Test’ datasets (see the text), respectively.

**parameter**	**value**
learning_rate	0.1
max_depth	2
min_samples_leaf	10
n_estimators	50 000
random_state	100
warm_start	TRUE

For prediction, the test example was first run through the GBC ‘gatekeeper’ to identify the class (‘maximum outliers’, ‘minimum outliers’ or ‘normal’) and the example was then run though the corresponding regression model.

### Cross validation

As an alternative test of the performance, 10-fold cross validation was performed using the scikitlearn module ‘RepeatedKFold’ on the larger ‘Nonredundant-New’ dataset and with features and hyperparameters from GBR4. For each of the 10-folds, hyperparameters remained unchanged, but each time the model would be retrained on 90% of the data and tested on the remaining 10%.

### Testing on an independent validation set

To validate results, the tests of the trained models were repeated on the independent validation set. The GBR4 model was retrained on the full ‘Nonredundant’ dataset (i.e. ‘Training’ plus ‘Test’) tested on the ‘Validation’ set. For the classification-regression approach, the models were not retrained before testing on the independent validation set.

### Extracting angles from AlphaFold2 generated models

Antibody models were generated for sequences (listed in Supplementary File ‘AF2-test.txt’) using the AlphaFold2 software. Since this evaluation was performed before AlphaFold-Multimer was available, the software only takes single protein chains. Consequently the V_H_ and V_L_ domains were linked using a (Gly$_4$Ser)$_n$ linker to form a single-chain Fv (scFv). ‘abpackingangle’ was then used to calculate the packing angle of the AlphaFold2 models.

## Results and Discussion

Antibody structure data available up until September 2022 were used in our experiments. The data were divided into train, test and independent validation sets as described in the Methods, and Supplementary Material.

### Predict Antibody Packing Angle

**Fig. 3:**
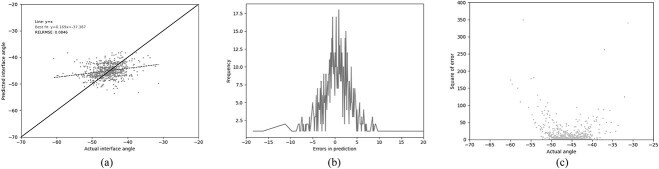
Performance of the original PAPA method on the new test set. (a) The relationship between the predicted and the actual angle. (b) The distribution of errors in packing angle prediction. (c) The squared error for each angle.

Abhinandan and Martin’s PAPA method (Abhinandan & Martin, 2010) was used as a basis for comparisons. The results of using the PAPA model to predict the packing angles in the test set are shown in Fig. 3. While Abhinandan and Martin obtained very good performance (RELRMSE=0.056; see Abhinandan and Martin (2010) for the definition of RELRMSE), Fig. 3(a) shows that, with the new test set, the model tends to predict values close to the mean ($\sim $-45${}^{\circ }$) regardless of the true angle and, as shown in Table 3 (PAPA), on this larger independent test set, the RELRMSE increases to 0.085. With a Pearson’s R of 0.244, the Abhinandan and Martin model is only slightly better than predicting the mean value in all cases.

**Table 3:** Summary of the main metrics for the combinations of methods and features that were tested; the ‘method’ is an identifier used to refer to the approach in the text; training and test sets are described in the Methods; ‘Residues’ refers to the number of residues used as features; for models using 13 residues, the positions are L38, L40, L41, L44, L46, L87, H33, H42, H45, H60, H62, H91 and H105, as used by Abhinandan and Martin (2010) in the original PAPA predictor; for models using 37 residues, the positions are L32, L34, L36, L38, L40, L41, L43, L44, L46, L50, L86, L87, L89, L91, L96, L98, H33, H35, H39, H42, H45, H47, H50, H60, H62, H91, H99, H100, H100A, H100B, H100C, H100D, H100E, H100F, H100G, H103 and H105; where ‘Loop lengths’ is ‘yes’, the lengths of CDR-L1, CDR-H2 and CDR-H3 were used as additional features; optimal models will have ‘Pearson’s R’ and ‘Slope’ close to 1, with all other metrics close to zero.

	Training	Test		Loop		Mean					
Method	set	set	Residues	lengths	Pearson’s R	error	RMSE	RELRMSE	Slope	Intercept	Figure no.
PAPA	Original PAPA	Test	13	no	0.244	2.843	3.871	0.085	0.169	−37.387	3
GBR4-P	Original PAPA	Test	37	yes	0.316	2.825	3.786	0.083	0.242	−34.269	S1
PAPA-Retrained	Training	Test	13	no	0.244	2.844	3.874	0.085	0.168	−37.408	S2
GBR1	Training	Test	13	no	0.672	1.920	2.688	0.059	0.523	−21.871	4
GBR2	Training	Test	13	yes	0.685	1.884	2.643	0.058	0.534	−21.351	5
GBR3	Training	Test	37	no	0.813	1.427	2.142	0.047	0.766	−10.731	6
GBR4	Training	Test	37	yes	0.816	1.433	2.126	0.046	0.766	−10.767	7
GBC/GBR	Training	Test	37	yes	0.807	1.425	2.185	0.048	0.770	−10.512	S3
GBR-X	Nonredundant-New	x-val	37	yes	0.780	1.498	2.380	0.052	0.687	−14.370	8
GBR-V	Training	Validation	37	yes	0.585	2.385	3.265	0.070	0.529	−21.226	9
GBC/GBR-V	Training	Validation	37	yes	0.567	2.294	3.243	0.070	0.474	−23.772	S4
AF2	AlphaFold2	AF2-test	N/A	N/A	0.371	2.766	3.952	0.086	0.161	−36.760	10
IgFold	IgFold	IgFold-test	N/A	N/A	0.164	2.322	4.232	0.092	0.180	−38.670	S5
MLP	Training	Test	37	yes	0.534	2.293	3.109	0.068	0.331	−31.149	S6

### Retrained PAPA

As a result, we retrained the PAPA neural network using the much larger ‘Training’ dataset described in the Methods and using the same larger test set. Surprisingly, the results were marginally worse than the original PAPA. See Table 3 (PAPA-Retrained) and Supplementary Figure S2. We therefore decided to explore different machine learning approaches.

### Gradient boosted regression

**Fig. 4:**
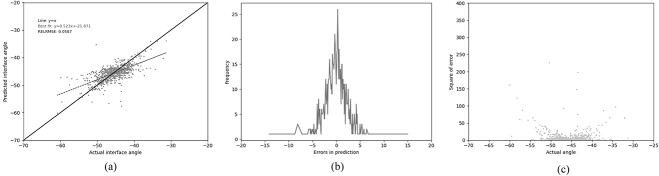
Performance of the GBR model (GBR1) trained on our ‘Training’ set and tested on out ‘Test’ set using the 13 residue feature set (see legend to Table 3). (a) The relationship between the predicted and the actual angle. (b) The distribution of errors in packing angle prediction. (c) The squared error for each angle.

**Fig. 5:**
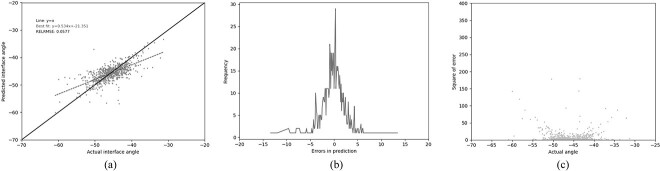
Performance of the GBR model (GBR2) trained on our ‘Training’ set and tested on out ‘Test’ set using the 13 residue feature set (see legend to Table 3) together with the lengths of CDR-L1, CDR-H2 and CDR-H3. (a) The relationship between the predicted and the actual angle. (b) The distribution of errors in packing angle prediction. (c) The square error for each angle.

In an attempt to improve the performance, the best performance was achieved using gradient boosted regression (GBR). The same 13 residues used by Abhinandan and Martin were employed, together with the same four-physical-parameter encoding. A huge improvement in performance was observed, with the Pearson’s correlation coefficient increasing to 0.672 and the RELRMSE dropping to 0.059 (Table 3 (GBR1), Fig. 4). In an attempt to improve the model further, the lengths of CDR-L1, CDR-H2 and CDR-H3 (the CDRs which vary most in length) were used as additional features. This resulted in a small improvement in performance, both in better correlation and lower error metrics (Table 3 (GBR2), Fig. 5).

### GBR with 37 residues

**Fig. 6:**
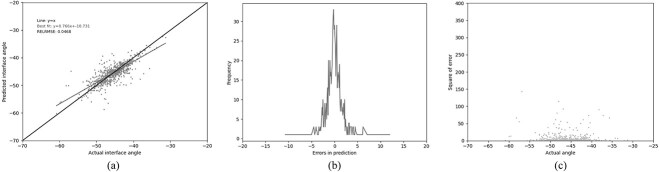
Performance of the GBR model (GBR3) trained on our ‘Training’ set and tested on our ‘Test’ set using the 37 residue feature set (see legend to Table 3). (a) The relationship between the predicted and the actual angle. (b) The distribution of errors in packing angle prediction. (c) The squared error for each angle.

**Fig. 7:**
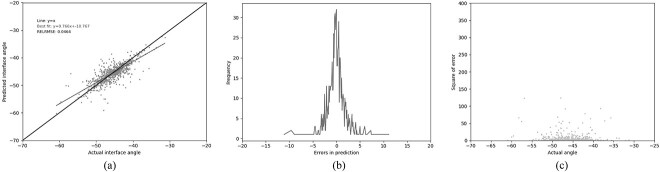
Performance of the GBR model (GBR4) trained on our ‘Training’ set and tested on our ‘Test’ set using the 37 residue feature set (see legend to Table 3) together with the lengths of CDR-L1, CDR-H2 and CDR-H3. (a) The relationship between the predicted and the actual angle. (b) The distribution of errors in packing angle prediction. (c) The square error for each angle.

As described above, the small size of the dataset available at the time of Abhinandan and Martin’s publication meant that the size of the training set was limited and the number of input features in the neural network had to be restricted. A genetic algorithm was used to limit the system to use the 13 most informative residues. However, since the data available are now over 20 times larger, there is room for adding potentially beneficial additional features. Consequently all 37 residues identified by Abhinandan and Martin as likely to influence the packing angle (prior to using the genetic algorithm to select the most influential positions) were used as features. These additional residues again dramatically improved the metrics when compared with PAPA, and show significant improvement when compared with using GBR with the 13 original residues (Table 3 (GBR3), Fig. 6.) Adding the loop lengths as three additional features also provides a small improvement in the performance, as it did with the smaller residue set. This provided the best GBR performance with the Pearson’s coefficient reaching a maximum compared with all other combinations tested, and the RELRMSE its minimum (Table 3 (GBR4), Fig. 7). To test the effect of training set size, we also trained a GBR model with 37 residues and loop lengths as features (equivalent to GBR4) using the same dataset on which the PAPA model was trained. The predictions on the test set showed that the model was essentially making random guesses around the mean (Table 3 (GBR4-P), Supplementary Figure S1). This clearly demonstrates that it is both the much larger training set and the use of GBR that improves the performance.

### Multi-layer perceptron

The original PAPA software was a standard artificial neural network multi-layer perceptron (MLP). While retraining the original PAPA using the larger dataset (but the same set of features) did not improve the performance, we decided to look at using an MLP with the expanded feature set (37 residues plus loop lengths) as a comparison with the more modern GBR methods.

For this implementation we used scikitlearn’s `MLPRegressor' with default hyperparameters, with the exception of hidden layer size adjusted to 15 and the maximum iterations set to 1200. The architecture was comprised of an input layer with 151 inputs (37 residues positions, encoded by four vectors, plus three loop lengths), one hidden layer with 15 neurons and an output layer with one neuron for the final angle. Other parameters were set to default (notably using activation function ‘ReLu’ and an alpha value of 0.0001). The MLP yielded a Pearson’s R of 0.534 and RELRMSE of 0.069, which is considerably better than the retrained PAPA (with fewer features), but not as good as GBR4 on the same training and testing sets. (Table 3 (MLP), Supplementary Figure S6).

### Combining regression and classification

Although the results achieved by the GBR model were very good, some of the outliers can be observed to have poorer predictions. In an attempt to mitigate this, a two-step method was developed, in which a GBC acts as a ‘gatekeeper’ to funnel the sequence into one of three regression models trained specifically on outliers or ‘normal’ values (-40${}^{\circ }$ to -50${}^{\circ }$) in the hope of improving outlier performance. However, the performance metrics were marginally worse than the GBR4 model (Table 3 (GBC/GBR), Supplementary Figure S3). Thus, including a classifier increases the computational cost, but produced no significant improvement in performance. The Matthews’ Correlation Coefficient for the GBC classification step was 0.620 (with 743 out of 816 test samples classified correctly, i.e. an accuracy of 0.911).

### Verification

**Fig. 8:**
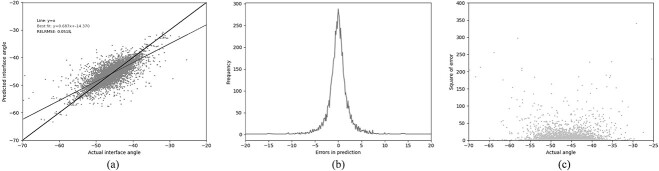
Performance of the GBR model (GBR-X) using 10-fold cross-validation on the full larger ‘Nonredundantnew’ dataset (i.e. the ‘Training’ plus ‘Test’ datasets) using the 37 residue feature set (see legend to Table 3) together with the lengths of CDR-L1, CDR-H2 and CDR-H3. (a) The relationship between the predicted and the actual angle. (b) The distribution of errors in packing angle prediction. (c) The squared error for each angle.

In all cases described above, a fixed training set and test set were used. To confirm that there was no bias between the train and test sets resulting from human optimization of parameters of the machine learning for the fixed test set, 10-fold cross-validation was performed using the full larger ‘Nonredundant-New’ set and the features used in GBR4. The performance only decreased a small amount from the optimum results with the GBR4 model test set (Table 3 (GBR-X), Fig. 8). The distribution around the predicted=actual line in Fig. 8(a) remains tight and most angle outliers are predicted within a range of 5${}^{\circ }$.

**Fig. 9:**
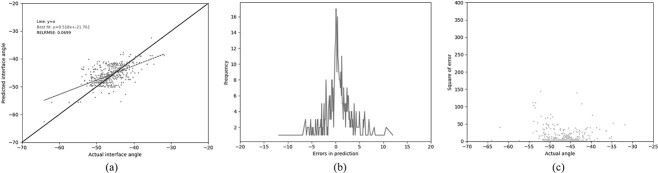
Performance of the GBR model (GBR-V) on an independent validation set using the 37 residue feature set (see legend to Table 3) together with the lengths of CDR-L1, CDR-H2 and CDR-H3. (a) The relationship between the predicted and the actual angle. (b) The distribution of errors in packing angle prediction. (c) The square error for each angle.

As a further validation of the performance, and to determine whether the hyperparameter tuning had overly favored the ‘Test’ set, the performance on the independent validation set (completely separate from the training and test sets used elsewhere) was evaluated. The GBR4 model was retrained on the whole ‘Nonredundant’ dataset (i.e. the combination of ‘Training’ and ‘Test’ sets) and tested on the independent validation set. As seen in Table 3 (GBR-V) and Fig. 9, the performance of the model was not as good as that of the GBR4 model, but it was still good. The independent validation set also confirmed no improvement in angle prediction when using a classifier in addition to the regressor (Table 3 (GBC/GBR-V), Supplementary Figure S4).

### AlphaFold2 and IgFold comparison

Recently, AlphaFold2 (Jumper *et al.*, 2021) has become the state of the art in general protein modeling. However, AlphaFold2 relies on the evolutionary information gained from multiple sequence alignment and its performance on antibodies is questionable because of the unique manner in which the affinity and specificity are refined through somatic hyper-mutation. To assess the performance of AlphaFold2 in predicting the V_H_/V_L_ packing, we built models using the standard AlphaFold2 (AlphaFold-Multimer was not available at the time this work was performed) on a subset of the available known structures (listed in Supplementary File ‘AF2-test.txt’) and compared these with our predictors.

**Fig. 10:**
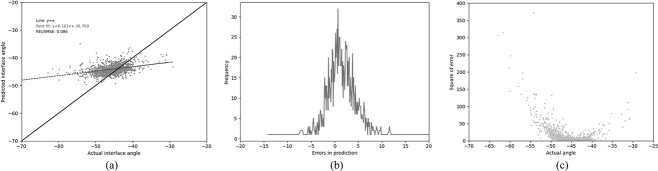
Performance of AlphaFold2 (AF2) in predicting the packing angle of antibodies. (a) The relationship between the predicted and the actual angle. (b) The distribution of errors in packing angle prediction. (c) The squared error for each angle.

The results (Table 3 (AF2) and Fig. 10) show that AlphaFold2 is relatively poor at predicting antibody V_H_/V_L_ packing angles. The RELRMSE (0.086) is higher than all other methods tried (including the original PAPA), while the Pearson’s R is 0.371. This is better than PAPA and PAPA-Retrained, but significantly worse than any of the methods which used GBR (Table 3).

We have also looked at the results obtained using IgFold (Ruffolo *et al.*, 2023). IgFold is a deep learning method which uses a pre-trained language model (trained on natural antibody sequences). After calculating the packing angles within the models built by IgFold, we observed that it tends to predict packing angles at around the mean of 48°. The Pearson’s R is 0.164, while the RELRMSE is 0.915, which is higher than the other methods we looked at (Table 3 (IgFold), Supplementary Figure S5).

## Conclusion

GBR provides a significant improvement from the simple feed-forward neural network used in PAPA. Further improvements are achieved by exploiting the larger available datasets which allow the use of additional features in machine learning. Our best predictor (GBR4), which we now refer to as abYpap, uses 40 features (37 residue positions and three loop lengths). All the methods tested are better than AlphaFold2 at predicting the packing angle. In addition we compared our results with those presented by Bujotzek *et al.* (2015). The most comparable of their parameters (HL) was predicted with an RMSE of 2.64 (compared with our RMSE of 2.13 for GBR4). However, our packing angle and HL cannot be compared directly as they describe slightly different angles and the datasets used are significantly different. It should also be noted that abYpap is a stand-alone easy-to-use open-source predictor, while Bujotzek *et al.*’s predictor is part of a larger modeling system.

As with our previous work, the applications of this work are 2-fold. First, we have shown that increasing the set of V_H_/V_L_ interface residues used increases the performance of the predictor. This implies that these additional residues are important in defining the packing angle and therefore may also be of importance in the antibody humanization. Second, this much-improved ability to predict the packing angle may be useful in improving the antibody modeling: the packing angle can be imposed on the model or used in selecting a single parent structure with the light and heavy chains paired in the correct orientation.

## Supplementary Material

supplementary_gzad021

Nonredundant-New_gzad021

AF2-test_gzad021

encoding_gzad021

ValidationSet_gzad021

## Software availability

abYpap is available from www.bioinf.org.uk/software/abypap/
