# Supplementary material for: abYpap: improvements to the prediction of antibody VH/VL packing using gradient boosted regression
Source: Protein Eng Des Sel. 2023 Nov 28;36:gzad021. doi: 10.1093/protein/gzad021 (PMC10719492; doi:10.1093/protein/gzad021)
Supplement: supplementary_gzad021 [file supplementary_gzad021.pdf]

Improvements to the Prediction of Antibody V_H/V_L Packing Using Gradient Boosted Regression Supplementary Figures

Veronica A. Boron and Andrew C.R. Martin

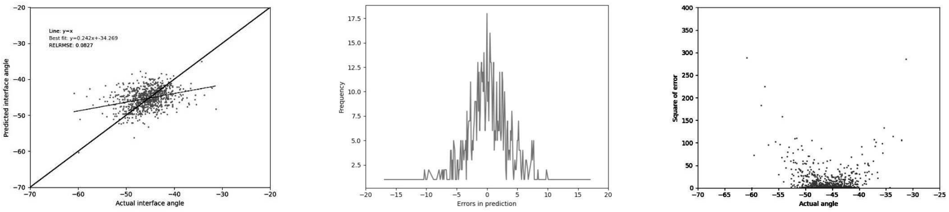

(a) (b) (c)

Figure S1: Results of training a gradient boosted model on original PAPA dataset and testing on 'Test'. (a) The relationship between the predicted and the actual angle. (b) The distribution of errors in packing angle prediction. (c) The squared error for each angle.

Alt caption: Graphs showing (a) the predicted interface angle plotted against the actual interface angle with the best-fit line compared against the perfect 1:1 slope. (b) the distribution of errors in prediction angle (negative or positive) shown in degrees. (c) a scatter plot showing the square of the error in the predicted packing plotted against the actual packing angle. All angles are shown in degrees.

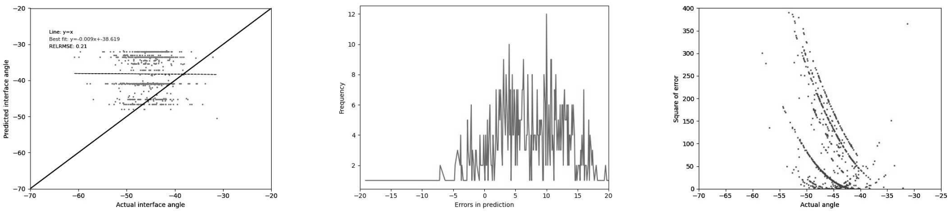

(a) (b) (c)

Figure S2: Results of retraining the original PAPA software on the new larger 'Training' dataset and testing on 'Test'. (a) The relationship between the predicted and the actual angle. (b) The distribution of errors in packing angle prediction. (c) The squared error for each angle.

Alt caption: Graphs showing (a) the predicted interface angle plotted against the actual interface angle with the best-fit line compared against the perfect 1:1 slope. (b) the distribution of errors in prediction angle (negative or positive) shown in degrees. (c) a scatter plot showing the square of the error in the predicted packing plotted against the actual packing angle. All angles are shown in degrees.

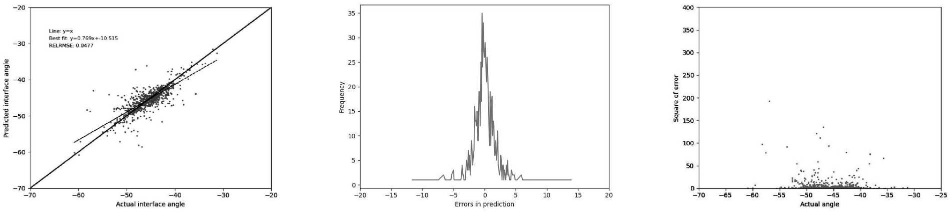

(a) (b) (c)

Figure S3: Performance of the Gradient Boosted Classifer gateway with three gradient boosted regression models (GBC/GBR) trained on our 'Training' set and tested on out 'Test' set using the 37 residue feature set (see legend to Table 1) together with the lengths of CDR-L1, CDR-H2 and CDR-H3. (a) The relationship between the predicted and the actual angle. (b) The distribution of errors in packing angle prediction. (c) The squared error for each angle.

Alt caption: Graphs showing (a) the predicted interface angle plotted against the actual interface angle with the best-fit line compared against the perfect 1:1 slope. (b) the distribution of errors in prediction angle (negative or positive) shown in degrees. (c) a scatter plot showing the square of the error in the predicted packing plotted against the actual packing angle. All angles are shown in degrees.

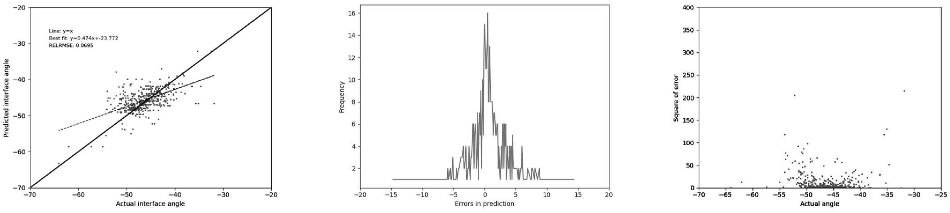

(a) (b) (c)

Figure S4: Performance of the gradient boosted classifer gateway with three gradient boosted regression models (GBC/GBR-V) when tested on the independent 'Validation' dataset. The model used the 37 residue feature set (see legend to Table 1) together with the lengths of CDR-L1, CDR-H2 and CDR-H3. (a) The relationship between the predicted and the actual angle. (b) The distribution of errors in packing angle prediction. (c) The squared error for each angle.

Alt caption: Graphs showing (a) the predicted interface angle plotted against the actual interface angle with the best-fit line compared against the perfect 1:1 slope. (b) the distribution of errors in prediction angle (negative or positive) shown in degrees. (c) a scatter plot showing the square of the error in the predicted packing plotted against the actual packing angle. All angles are shown in degrees.

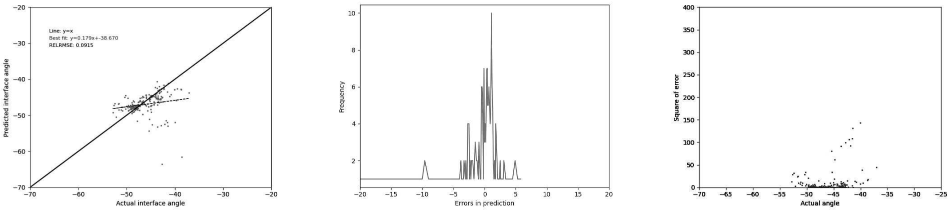

(a) (b) (c)

Figure S5: Performance of IgFold on a 300 sample dataset. (a) The relationship between the predicted and the actual angle. (b) The distribution of errors in packing angle prediction. (c) The squared error for each angle.

Alt caption: Graphs showing (a) the predicted interface angle plotted against the actual interface angle with the best-fit line compared against the perfect 1:1 slope. (b) the distribution of errors in prediction angle (negative or positive) shown in degrees. (c) a scatter plot showing the square of the error in the predicted packing plotted against the actual packing angle. All angles are shown in degrees.

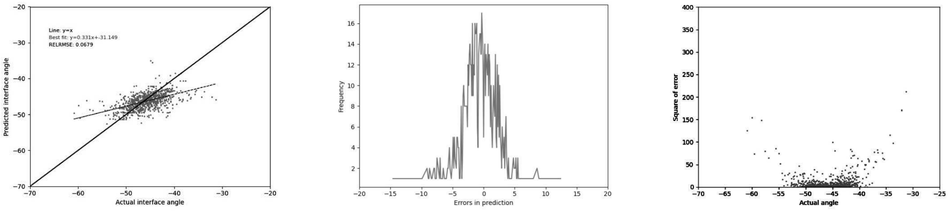

(a) (b) (c)

Figure S6: Performance of the multilayer perceptron gateway with when tested on the ‘Test’ dataset. The model used the 37 residue feature set (see legend to Table 1) together with the lengths of CDR-L1, CDR-H2 and CDR-H3. (a) The relationship between the predicted and the actual angle. (b) The distribution of errors in packing angle prediction. (c) The squared error for each angle.

Alt caption: Graphs showing (a) the predicted interface angle plotted against the actual interface angle with the best-fit line compared against the perfect 1:1 slope. (b) the distribution of errors in prediction angle (negative or positive) shown in degrees. (c) a scatter plot showing the square of the error in the predicted packing plotted against the actual packing angle. All angles are shown in degrees.
